# 2,2′-[1,1′-(Propane-1,3-diyldioxy­dinitrilo)diethyl­idyne]diphenol

**DOI:** 10.1107/S1600536808012701

**Published:** 2008-05-17

**Authors:** Wen-Kui Dong, Xue-Ni He, Jin-Kui Zhong, Xiao Chen, Tian-Zhi Yu

**Affiliations:** a School of Chemical and Biological Engineering, Lanzhou Jiaotong University, Lanzhou 730070, People’s Republic of China; b Key Laboratory of Opto-Electronic Technology and Intelligent Control, Ministry of Education, Lanzhou 730070, People’s Republic of China

## Abstract

The title compound, C_19_H_22_N_2_O_4_, was synthesized by the reaction of 2′-hydroxy­acetophenone with 1,3-bis­(amino­oxy)propane in ethanol. Intra­molecular O—H⋯N and weak C—H⋯O hydrogen bonds stabilize the three-dimensional structure. A twofold rotation axis passes through the molecule.

## Related literature

For related literature, see: Atkins *et al.* (1985 ▶); Atwood (1997 ▶); Costes *et al.* (2000 ▶); Dong & Feng (2006 ▶); Dong *et al.* (2006*a* ▶,*b* ▶, 2007*a* ▶,*b* ▶,*c* ▶,*d* ▶); Duan *et al.* (2007 ▶); Katsuki (1995 ▶); Lacroix (2001 ▶); Venkataramanan *et al.* (2005 ▶); Yu *et al.* (2008 ▶); Zhang *et al.* (2007 ▶).
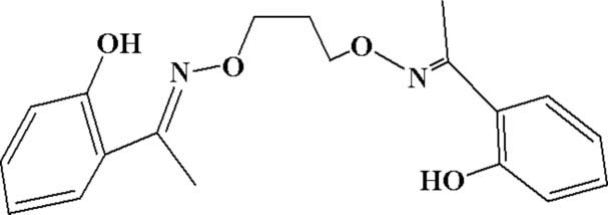


## Experimental

### 

#### Crystal data

C_19_H_22_N_2_O_4_
*M* *_r_* = 342.39
Orthorhombic, 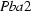

*a* = 7.4595 (15) Å
*b* = 25.459 (2) Å
*c* = 4.5938 (8) Å
*V* = 872.4 (2) Å^3^
*Z* = 2
Mo *K*α radiation
μ = 0.09 mm^−1^
*T* = 298 (2) K
0.40 × 0.19 × 0.17 mm

#### Data collection

Bruker SMART CCD area-detector diffractometer
Absorption correction: multi-scan (*SADABS*; Sheldrick, 1996 ▶) *T*_min_ = 0.964, *T*_max_ = 0.985
3761 measured reflections
880 independent reflections
601 reflections with *I* > 2σ(*I*)
*R*_int_ = 0.080

#### Refinement

*R*[*F* ^2^ > 2σ(*F* ^2^)] = 0.052
*wR*(*F* ^2^) = 0.162
*S* = 1.12
880 reflections
114 parameters
1 restraint
H-atom parameters constrained
Δρ_max_ = 0.17 e Å^−3^
Δρ_min_ = −0.20 e Å^−3^

### 

Data collection: *SMART* (Siemens, 1996 ▶); cell refinement: *SAINT* (Siemens, 1996 ▶); data reduction: *SAINT*; program(s) used to solve structure: *SHELXS97* (Sheldrick, 2008 ▶); program(s) used to refine structure: *SHELXL97* (Sheldrick, 2008 ▶); molecular graphics: *SHELXTL* (Sheldrick, 2008 ▶); software used to prepare material for publication: *SHELXTL*.

## Supplementary Material

Crystal structure: contains datablocks global, I. DOI: 10.1107/S1600536808012701/hg2386sup1.cif

Structure factors: contains datablocks I. DOI: 10.1107/S1600536808012701/hg2386Isup2.hkl

Additional supplementary materials:  crystallographic information; 3D view; checkCIF report

## Figures and Tables

**Table 1:** Hydrogen-bond geometry (Å, °)

*D*—H⋯*A*	*D*—H	H⋯*A*	*D*⋯*A*	*D*—H⋯*A*
O2—H2⋯N1	0.82	1.85	2.570 (5)	146
C3—H3*A*⋯O1	0.96	2.17	2.603 (6)	106

**Fig. 1.**
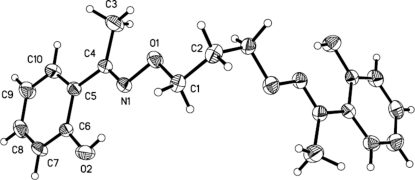
Molecule structure of (I) possessing a crystallographic twofold rotation axis passing through the middle point of the C—C—C unit (symmetry code: -x+1, -y, z), Displacement ellipsoids for non-hydrogen atoms are drawn at the 30% probability level.

## supplementary crystallographic information

### Comment

Salen-type compounds have been intensively used as versatile chelating ligands in the formation of transition metal complexes (Yu *et al.*, 2008). Some of them or their metal complexes are used in various organic reaction processes as catalysts (Venkataramanan *et al.*, 2005), models of reaction centers of metalloenzymes (Katsuki *et al.*, 1995), have fascinating magnetic properties (Costes *et al.*, 2000) and are nonlinear optical materials (Lacroix *et al.*, 2001). They can also be used as biological models in understanding the structure of biomolecules and biological processes (Atkins *et al.*, 1985, Atwood *et al.*, 1997). Most of their important features of these compounds are their preparative accessibility, diversity and structural variability, which make them more attractive.

In recent years, we have been very much interested in the synthesis and study of salen-type bisoxime derivatives, such as 2,2'-[(1,4-butylene)dioxybis(nitrilomethylidyne)]dinaphthol (Dong *et al.*, 2006*a*), 4,4'-dibromo-2,2'-[ethylenedioxybis(nitrilomethylidyne)]diphenol (Dong & Feng, 2006), 4,4'-dibromo-2,2'-[(1,3-propylene) dioxybis(nitrilomethylidyne)]diphenol (Dong *et al.*, 2006*b*), 2,2'-[(1,4-butylene)dioxybis(nitrilomethylidyne)]diphenol (Dong *et al.*, 2007*a*), 4,4'-dichloro-2,2'-[(1,4-butylene)dioxybis(nitrilomethylidyne)]diphenol (Dong *et al.*, 2007*b*), 4,4'6,6'-tetra(*tert*-butyl)-2,2'-[(1,4-butylene)dioxybis (nitrilomethylidyne)]diphenol (Dong *et al.*, 2007*c*), 2,2'-[(1,4-butylene)dioxybis(nitriloethylidyne)]diphenol (Dong *et al.*, 2007d), 2,2'-[(propane-1,3-diyldioxy)bis(nitrilomethylidyne)]diphenol (Duan *et al.*, 2007), and 5,5'-bis(diethylamino)-2,2'-[ethylenedioxybis(nitrilomethylidyne)]diphenol (Zhang *et al.*, 2007). In this paper, a novel bisoxime ligand, 2,2'-[(propane-1,3-diyldioxy)bis(nitriloethylidyne)]diphenol (I) was designed and synthesized, and shown in Fig. 1.

The single-crystal structure of (I) is built up by discrete C_19_H_22_N_2_O_4_ molecules (Fig. 1), in which all bond lengths are in normal ranges. There is a crystallographic twofold rotation axis passing through the middle point (symmetry code: -x, -y, z) of the C—C—C unit. The molecule adopts a trans conguration in which two phenoldoxime moieties adopts an extended form, where the oxime, methyl groups and phenolic alcohols lie in trans positions relative to the C2 atom in the N—-O—CH_2_—CH_2_—CH_2_—O—N linkage, which is similar to what is observed in our previously reported salen-type bisoxime of 2,2'-[(propane-1,3-diyldioxy)bis(nitrilomethylidyne)]diphenol (Duan *et al.*, 2007). There is an intramolecular O—H···N hydrogen bond between the N1 atom and the hydroxy proton (Table 1) generating a six membered ring, which with weak C—H···O intermolecular hydrogen bonds, stabilizes the three-dimensional structure of (I).

### Experimental

2,2'-[(Propane-1,3-diyldioxy)bis(nitriloethylidyne)]diphenol was synthesized according to an analogous method reported earlier (Dong *et al.*, 2007d). To an ethanol solution (5 ml) of 2'-hydroxyacetophenone (280.9 mg, 2.01 mmol) was added an ethanol (3 ml) solution of 1,3-bis(aminooxy)propane (105.5 mg, 1.00 mmol). The mixture solution was stirred at 328 K for 3 h. After cool to room temperature, the precipitate was formed, which was filtered, and washed successively with ethanol and ethanol/hexane (1:4), respectively. The product was dried under vacuum and to yield 64.90 mg of the title compound. Yield, 19.1%. mp. 363–363.5 K. Anal. Calc. for C_19_H_22_N_2_O_4_: C, 66.65; H, 6.48; N, 8.18. Found: C, 66.76; H, 6.39; N, 7.97. Colorless needle-shaped single crystals suitable for X-ray diffraction studies were obtained after three months by slow evaporation from an ethanol solution (10 ml) of 2,2'-[(propane-1,3-diyldioxy)bis(nitriloethylidyne)]diphenol.

### Refinement

H atoms were treated as riding atoms with distances C—H = 0.97 (CH_2_), or 0.93 Å (CH),*O*—H = 0.82 Å, and *U*_iso_(H) = 1.2 *U*_eq_(C) and 1.5 *U*_eq_(O). The hydroxyl protons were located directly from a Fourier map.

### Crystal data

**Table:**

C_19_H_22_N_2_O_4_	*F*_000_ = 364
*M**_r_* = 342.39	*D*_x_ = 1.303 Mg m^−^^3^
Orthorhombic, *P**b**a*2	Mo *K*α radiation λ = 0.71073 Å
Hall symbol: P 2 -2ab	Cell parameters from 1047 reflections
*a* = 7.4595 (15) Å	θ = 2.4–22.9º
*b* = 25.459 (2) Å	µ = 0.09 mm^−^^1^
*c* = 4.5938 (8) Å	*T* = 298 (2) K
*V* = 872.4 (2) Å^3^	Needle-shaped, colorless
*Z* = 2	0.40 × 0.19 × 0.17 mm

### Data collection

**Table:**

Bruker SMART CCD area-detector diffractometer	880 independent reflections
Radiation source: fine-focus sealed tube	601 reflections with *I* > 2σ(*I*)
Monochromator: graphite	*R*_int_ = 0.080
*T* = 298(2) K	θ_max_ = 25.0º
φ and ω scans	θ_min_ = 1.6º
Absorption correction: multi-scan(SADABS; Sheldrick, 1996)	*h* = −8→4
*T*_min_ = 0.964, *T*_max_ = 0.985	*k* = −30→28
3761 measured reflections	*l* = −5→5

### Refinement

**Table:**

Refinement on *F*^2^	Secondary atom site location: difference Fourier map
Least-squares matrix: full	Hydrogen site location: inferred from neighbouring sites
*R*[*F*^2^ > 2σ(*F*^2^)] = 0.052	H-atom parameters constrained
*wR*(*F*^2^) = 0.162	*w* = 1/[σ^2^(*F*_o_^2^) + (0.09*P*)^2^] where *P* = (*F*_o_^2^ + 2*F*_c_^2^)/3
*S* = 1.12	(Δ/σ)_max_ < 0.001
880 reflections	Δρ_max_ = 0.18 e Å^−^^3^
114 parameters	Δρ_min_ = −0.20 e Å^−^^3^
1 restraint	Extinction correction: none
Primary atom site location: structure-invariant direct methods	

### Special details

**Table:**

Geometry. All e.s.d.'s (except the e.s.d. in the dihedral angle between two l.s. planes) are estimated using the full covariance matrix. The cell e.s.d.'s are taken into account individually in the estimation of e.s.d.'s in distances, angles and torsion angles; correlations between e.s.d.'s in cell parameters are only used when they are defined by crystal symmetry. An approximate (isotropic) treatment of cell e.s.d.'s is used for estimating e.s.d.'s involving l.s. planes.
Refinement. Refinement of *F*^2^ against ALL reflections. The weighted *R*-factor *wR* and goodness of fit *S* are based on *F*^2^, conventional *R*-factors *R* are based on *F*, with *F* set to zero for negative *F*^2^. The threshold expression of *F*^2^ > σ(*F*^2^) is used only for calculating *R*-factors(gt) *etc*. and is not relevant to the choice of reflections for refinement. *R*-factors based on *F*^2^ are statistically about twice as large as those based on *F*, and *R*- factors based on ALL data will be even larger.

### Fractional atomic coordinates and isotropic or equivalent isotropic displacement parameters (Å^2^)

**Table:**

	*x*	*y*	*z*	*U*_iso_*/*U*_eq_	Occ. (<1)
N1	0.7665 (5)	0.07883 (13)	0.2794 (9)	0.0424 (10)	
O1	0.6235 (4)	0.06405 (11)	0.0997 (8)	0.0497 (10)	
O2	1.0675 (4)	0.06514 (11)	0.5410 (10)	0.0613 (12)	
H2	0.9857	0.0579	0.4284	0.092*	
C1	0.6621 (6)	0.01443 (16)	−0.0329 (12)	0.0447 (13)	
H1A	0.6845	−0.0120	0.1148	0.054*	
H1B	0.7671	0.0171	−0.1564	0.054*	
C2	0.5000	0.0000	−0.2107 (16)	0.0479 (18)	
H2A	0.5305	−0.0294	−0.3353	0.058*	0.50
H2B	0.4695	0.0294	−0.3353	0.058*	0.50
C3	0.5696 (7)	0.15361 (18)	0.3585 (17)	0.0660 (17)	
H3A	0.5088	0.1406	0.1891	0.099*	
H3B	0.5989	0.1900	0.3308	0.099*	
H3C	0.4931	0.1500	0.5254	0.099*	
C4	0.7390 (6)	0.12265 (17)	0.4060 (10)	0.0406 (12)	
C5	0.8802 (6)	0.14197 (16)	0.5999 (11)	0.0380 (11)	
C6	1.0350 (6)	0.11254 (15)	0.6663 (11)	0.0396 (12)	
C7	1.1585 (6)	0.1310 (2)	0.8622 (13)	0.0540 (15)	
H7	1.2586	0.1107	0.9065	0.065*	
C8	1.1363 (6)	0.1787 (2)	0.9934 (16)	0.0582 (15)	
H8	1.2207	0.1908	1.1263	0.070*	
C9	0.9884 (8)	0.2086 (2)	0.9277 (16)	0.0649 (18)	
H9	0.9733	0.2414	1.0135	0.078*	
C10	0.8645 (7)	0.19013 (18)	0.7369 (13)	0.0521 (15)	
H10	0.7646	0.2108	0.6966	0.063*	

### Atomic displacement parameters (Å^2^)

**Table:**

	*U*^11^	*U*^22^	*U*^33^	*U*^12^	*U*^13^	*U*^23^
N1	0.038 (2)	0.047 (2)	0.043 (2)	−0.0020 (17)	−0.005 (2)	0.002 (2)
O1	0.0458 (18)	0.0516 (19)	0.052 (2)	−0.0039 (14)	−0.0123 (19)	−0.0063 (18)
O2	0.053 (2)	0.054 (2)	0.077 (3)	0.0138 (15)	−0.018 (2)	−0.0045 (19)
C1	0.048 (3)	0.041 (2)	0.045 (3)	−0.004 (2)	0.005 (3)	−0.001 (2)
C2	0.068 (5)	0.046 (3)	0.030 (4)	−0.007 (3)	0.000	0.000
C3	0.051 (3)	0.060 (3)	0.087 (5)	0.009 (2)	−0.021 (4)	−0.015 (3)
C4	0.038 (2)	0.042 (2)	0.042 (3)	0.000 (2)	−0.005 (2)	0.004 (2)
C5	0.035 (2)	0.045 (2)	0.034 (3)	−0.001 (2)	−0.001 (2)	0.004 (2)
C6	0.035 (2)	0.042 (2)	0.042 (3)	−0.002 (2)	−0.002 (2)	0.010 (2)
C7	0.036 (3)	0.066 (3)	0.060 (4)	0.002 (2)	−0.015 (3)	0.009 (3)
C8	0.046 (3)	0.072 (3)	0.057 (4)	−0.015 (3)	−0.011 (3)	−0.001 (3)
C9	0.057 (3)	0.056 (3)	0.082 (5)	−0.003 (3)	−0.017 (4)	−0.016 (3)
C10	0.043 (3)	0.056 (3)	0.057 (4)	0.006 (2)	−0.004 (3)	0.003 (3)

### Geometric parameters (Å, °)

**Table:**

N1—C4	1.275 (5)	C3—H3B	0.9600
N1—O1	1.400 (5)	C3—H3C	0.9600
O1—C1	1.432 (5)	C4—C5	1.464 (6)
O2—C6	1.359 (5)	C5—C10	1.383 (6)
O2—H2	0.8200	C5—C6	1.410 (6)
C1—C2	1.505 (6)	C6—C7	1.370 (7)
C1—H1A	0.9700	C7—C8	1.366 (7)
C1—H1B	0.9700	C7—H7	0.9300
C2—C1^i^	1.505 (6)	C8—C9	1.374 (7)
C2—H2A	0.9700	C8—H8	0.9300
C2—H2B	0.9700	C9—C10	1.358 (8)
C3—C4	1.505 (6)	C9—H9	0.9300
C3—H3A	0.9600	C10—H10	0.9300
			
C4—N1—O1	112.4 (3)	N1—C4—C5	117.1 (4)
N1—O1—C1	109.5 (3)	N1—C4—C3	121.8 (4)
C6—O2—H2	109.5	C5—C4—C3	121.1 (4)
O1—C1—C2	106.5 (3)	C10—C5—C6	116.2 (4)
O1—C1—H1A	110.4	C10—C5—C4	120.9 (4)
C2—C1—H1A	110.4	C6—C5—C4	122.8 (4)
O1—C1—H1B	110.4	O2—C6—C7	117.6 (4)
C2—C1—H1B	110.4	O2—C6—C5	121.7 (4)
H1A—C1—H1B	108.6	C7—C6—C5	120.7 (4)
C1—C2—C1^i^	114.3 (6)	C8—C7—C6	120.8 (5)
C1—C2—H2A	108.7	C8—C7—H7	119.6
C1^i^—C2—H2A	108.7	C6—C7—H7	119.6
C1—C2—H2B	108.7	C7—C8—C9	119.6 (5)
C1^i^—C2—H2B	108.7	C7—C8—H8	120.2
H2A—C2—H2B	107.6	C9—C8—H8	120.2
C4—C3—H3A	109.5	C10—C9—C8	119.7 (5)
C4—C3—H3B	109.5	C10—C9—H9	120.1
H3A—C3—H3B	109.5	C8—C9—H9	120.1
C4—C3—H3C	109.5	C9—C10—C5	122.9 (5)
H3A—C3—H3C	109.5	C9—C10—H10	118.5
H3B—C3—H3C	109.5	C5—C10—H10	118.5
			
C4—N1—O1—C1	−179.4 (4)	C4—C5—C6—O2	3.6 (7)
N1—O1—C1—C2	177.5 (4)	C10—C5—C6—C7	1.7 (7)
O1—C1—C2—C1^i^	−70.3 (3)	C4—C5—C6—C7	−176.2 (4)
O1—N1—C4—C5	180.0 (3)	O2—C6—C7—C8	178.9 (5)
O1—N1—C4—C3	−0.3 (7)	C5—C6—C7—C8	−1.3 (8)
N1—C4—C5—C10	177.4 (5)	C6—C7—C8—C9	−0.1 (9)
C3—C4—C5—C10	−2.3 (7)	C7—C8—C9—C10	1.1 (10)
N1—C4—C5—C6	−4.9 (6)	C8—C9—C10—C5	−0.7 (9)
C3—C4—C5—C6	175.4 (5)	C6—C5—C10—C9	−0.7 (8)
C10—C5—C6—O2	−178.6 (4)	C4—C5—C10—C9	177.2 (5)

Symmetry codes: (i) −*x*+1, −*y*, *z*.

### Hydrogen-bond geometry (Å, °)

**Table:**

*D*—H···*A*	*D*—H	H···*A*	*D*···*A*	*D*—H···*A*
O2—H2···N1	0.82	1.85	2.570 (5)	146
C3—H3A···O1	0.96	2.17	2.603 (6)	106
